# Antifungal potential of *Bacillus* strains: implications for biocontrol strategies in food safety and sustainable agriculture

**DOI:** 10.3389/fmicb.2025.1615252

**Published:** 2025-07-15

**Authors:** Houda Gharsallah, Manel Cheffi, Rahma Mallek, Noura Omri, Mohamed Ali Triki, Mecit Halil Öztop, Zied Zarai

**Affiliations:** ^1^Laboratory of Improvement and Protection of Olive Tree Genetic Resources, Olive Tree Institute, University of Sfax, Sfax, Tunisia; ^2^Department of Food Technology, High Institute of Biotechnology of Sfax, University of Sfax, Sfax, Tunisia; ^3^Field Crop Laboratory, National Institute for Agricultural, Research of Tunisia (INRAT), Tunis, Tunisia; ^4^Faculty of Engineering, Food Engineering, Middle East Technical University, Ankara, Türkiye; ^5^Laboratory of Biochemistry and Enzymatic Engineering of Lipases, ENIS, University of Sfax, Sfax, Tunisia

**Keywords:** biosurfactants, lipopeptides, *Bacillus*, fungal food spoilage, antifungal activity, volatile organic compounds (VOCs)

## Abstract

**Introduction:**

Microbial spoilage and fungal phytopathogen infections significantly reduce the shelf life of perishable foods, creating major challenges for both agriculture and food supply chains. *Bacillus* species are known producers of antifungal metabolites such as lipopeptides and volatile organic compounds (VOCs), offering a sustainable alternative to synthetic fungicides.

**Methods:**

This study evaluated the antagonistic activity of four *Bacillus* strains, H6 (*Bacillus velezensis*), S15 and S40 (*Bacillus subtilis*), and S32 (*Bacillus cereus*) against nine fungal phytopathogens, including those affecting tomatoes. Assessment methods included 108 dual-culture assays, *in vitro* lipopeptide bioassays, VOC-mediated inhibition tests, and PCR screening for genes involved in lipopeptide biosynthesis.

**Results:**

Strains H6 and S15 exhibited strong antagonistic effects, inhibiting mycelial growth by up to 78% for *Botrytis cinerea* (H6) and 87% for S15. Lipopeptide bioassays revealed that purified extracts from H6, S15, and S32 inhibited *Rhizoctonia solani* mycelial growth by 60%. VOCs produced by all four strains completely inhibited the growth of *Fusarium oxysporum* and *Lasiodiplodia theobromae*, with strain S40 showing the strongest VOC-mediated inhibition.

**Discussion:**

These results demonstrate the significant antifungal potential of Bacillus strains H6 and S15, which produce iturin/fengycin-type lipopeptides and VOCs, as supported by PCR detection of biosynthetic genes. These strains represent promising candidates for environmentally friendly strategies in food preservation and agricultural biocontrol.

## Graphical abstract

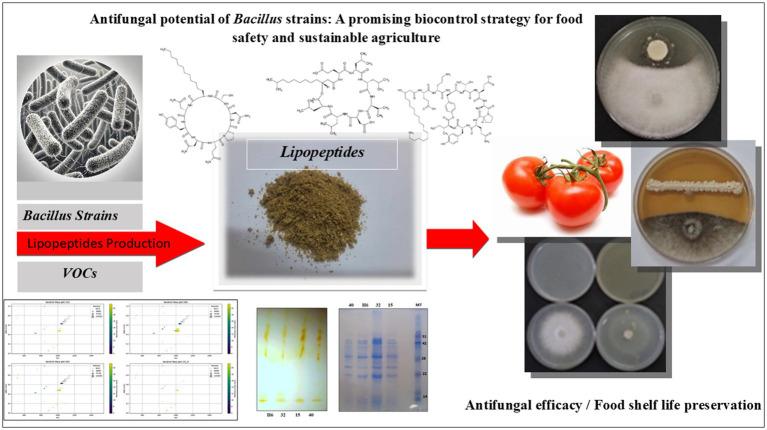

## 1 Introduction

Extending the shelf life of perishable foods remains a critical challenge in agriculture and food supply chains, primarily due to microbial spoilage and phytopathogen infections. These issues lead to significant economic losses and threaten global food security (FAO, 2019). To address this, biological control strategies using beneficial microorganisms have emerged as sustainable alternatives to synthetic preservatives and chemical pesticides (Liu et al., 2024; An et al., 2024). Lipopeptides, as biosurfactants, are bioactive molecules composed of a lipid moiety linked to a peptide chain. They are synthesized by various organisms, including mammals, fungi, bacteria, and plants (Biniarz et al., 2017). These compounds possess notable therapeutic properties and biological roles, including the ability to lower surface tension, interfere with quorum sensing, and exhibit antimicrobial effects against various pathogens (Meena and Kanwar, 2015; Segovia et al., 2021).

Lipopeptides exhibit potent activity against a wide range of pathogens and are considered promising scaffolds for developing antibiotics targeting multidrug-resistant bacteria. Among microbial producers, Bacillus species, particularly *Bacillus subtilis* and *Bacillus amyloliquefaciens*, are known for synthesizing beneficial lipopeptides with applications in both agriculture and biomedicine (Liu et al., 2024; An et al., 2024; Segovia et al., 2021; Sreedharan et al., 2023).

The *Bacillus* genus is known to produce three primary families of lipopeptides: surfactin, iturin, and fengycin. These lipopeptides are effective in reducing surface tension and exhibit strong antimicrobial activity against various pathogens, including fungi (Biniarz et al., 2017; Théatre et al., 2021; Yuan et al., 2025). For example, surfactin is primarily antibacterial, while iturins and fengycins are more active against fungi (Meena and Kanwar, 2015). Differences in fatty acid chain length, peptide cyclization, and amino acid composition also play a role in their bioactivity. However, studies directly linking these structural features to antimicrobial potency are still limited (Ongena and Jacques, 2008). Moreover, much of the existing research has concentrated on individual lipopeptide families or specific pathogens, often neglecting the synergistic effects of naturally occurring lipopeptide mixtures in Bacillus strains (Caulier et al., 2019). For example, lipopeptides can inhibit biofilm formation in Gram-negative bacteria, a significant capability since biofilm-associated microbes are often more resistant to biocides. They also suppress virulence factors in pathogens; for instance, lipopeptides reduced the production of hemolysin A, a key toxin in *Staphylococcus aureus*—in a dose-dependent manner, with complete inhibition observed at 100 μg/mL (Wang D. et al., 2022; Sabino et al., 2024).

The primary mechanism of lipopeptides involves membrane disruption leading to cell lysis (Sabino et al., 2024). Their multi-target action, biodegradability, and low toxicity provide significant advantages over traditional antibiotics, making them suitable for combating antibiotic resistance (Ji et al., 2024; Ma et al., 2022). In agriculture, lipopeptide-based gels and microspheres hold potential for protecting crops, while in biomedicine, they are being explored for drug development through structural modifications to improve their effectiveness (Valenzuela et al., 2024; Yuan et al., 2025).

This study aims to assess the antifungal activity of lipopeptides produced by *Bacillus* strains against nine phytopathogenic fungi through direct antagonism assays. By also evaluating the effect of volatile organic compounds (VOCs), this work aims to enhance our understanding of their biocontrol potential and their role in extending food shelf life.

## 2 Materials and methods

### 2.1 Antagonistic bacteria

Bacterial strains were isolated from soil samples using heat treatment and spread plate methods on Luria Bertani (LB) agar (Gharsallah et al., 2025). Strains exhibiting hemolytic activity on Blood Agar (Gharsallah et al., 2025) were selected for further study. Identification of the *Bacillus* spp. strains (H6, S15, S32, and S40) was performed by 16S rRNA gene sequencing (Gharsallah et al., 2025). These strains were cultured in an optimized medium designed to enhance bioactive compound production and incubated at 30°C for 5 days with shaking. Lipopeptides were extracted from the culture supernatant by pH adjustment, precipitation, and lyophilization for subsequent analysis. The selected *Bacillus* strains demonstrated high lipopeptide production, making them suitable candidates for investigating their antimicrobial properties and potential applications in food preservation, crop protection, and sustainable biocontrol strategies.

### 2.2 Phytopathogenic fungi

Nine phytopathogenic fungi were used in this study, including *Fusarium oxysporum* (*F. oxy*), *Alternaria alternata* (*A. alt*), *Fusarium solani* (*F. sol*), *Fusarium oxysporum* f. sp. *radicis-lycopersici* (*FORL*), *Botrytis cinerea* (*B. cin*), *Verticillium dahliae* (*V. dah*), *Rhizoctonia solani* (*R. sol*), *Lasiodiplodia theobromae* (*L. the*), and *Rhizoctonia bataticola* (*R. bat*). The fungal pathogens tested in this study are highly relevant to agriculture, as they cause significant yield losses in tomatoes crops. The fungal pathogens were isolated from infected samples by surface sterilization and culturing on Potato Dextrose Agar. Pure cultures were obtained through subculturing and examined microscopically using Methylene Blue Staining to observe hyphal and spore morphology for preliminary identification. Molecular confirmation was achieved by extracting genomic DNA, amplifying the ITS region via PCR with universal primers (ITS1/ITS4), and sequencing the products. The sequences were compared against NCBI GenBank databases using BLASTn for species-level identification. For long-term storage, the fungal strains were maintained on potato dextrose agar plates at 4°C and in a tryptone salt medium (comprising 1 g/L tryptone, 8.5 g/L NaCl, 1% (v/v) Tween 20, and 15% (v/v) glycerol) at −20°C (Cheffi et al., 2020).

### 2.3 PCR detection of genes related to the biosynthesis of lipopeptides

Genes associated with the biosynthesis of lipopeptides, such as surfactin, fengycin, iturin, and bacillomycin, were identified using PCR with primers listed in Table 1 and following steps described by Ben Abdallah et al. (2015, 2018) and Cheffi et al. (2020). PCR reactions were performed in a 50 μL mixture containing 10 μL of 5 × PCR buffer, 4 μL of 25 mmol/L MgCl₂, 5 μL of dNTP mix (0.2 mmol/L), 5 μL each of forward and reverse primers (10 mmol/L), 2 U of Taq DNA polymerase (GoTaq), and 50 ng of template DNA. The thermal cycling protocol included an initial denaturation at 95°C for 5 min, followed by 30 cycles of denaturation at 95°C for 1 min, primer annealing at 50–58°C for 1 min, and extension at 72°C for 1.5 min. A final extension at 72°C for 7 min was performed to ensure complete amplification. This method has been extensively used and validated in prior studies (Ben Abdallah et al., 2015, 2018; Cheffi et al., 2020). The amplified PCR products were purified and sequenced using an automatic sequencer (Avant Genetic Analyzer, Model 3100). The PCR sequences were analyzed using the Basic Local Alignment Search Tool (BLAST) and the GenBank nucleotide database from the National Center for Biotechnology Information (NCBI) in Bethesda, MD, USA.^1^

**Table 1:** PCR primers used for the identification of genes involved in the biosynthesis of lipopeptides (surfactin, fengycin, iturin, and bacillomycin).

Lipopeptides	Genes	Primers	Sequences	PCR product size (pb)
Surfactin	Sfp	Sfp-F	5′-ATGAAGATTTACGGAATTTA-3′	675
		Sfp-R	5′-TTATAAAAGCTCTTCGTACG-3′	
	SrfaA	Srfa-F	5′-TCGGGACAGGAAGACATCAT-3′	201
		Srfa-R	5′-CCACTCAAACGGATAATCCTGA-3′	
Iturin	ItuD	ITUD-F1	5′-TTGAAYGTCAGYGCSCCTTT-3′	482
		ITUD-R1	5′-TGCGMAAATAATGGSGTCGT-3′	
	ItuC	ITUC-F1	5′-CCCCCTCGGTCAAGTGAATA-3′	594
		ITUC-R1	5′-TTGGTTAAGCCCTGATGCTC-3′	
Fengycin	FenB	FENB2F	5′-CAAGATATGCTGGACGCTGA-3′	964
		FENB2R	5′-ACACGACATTGCGATTGGTA-3′	
	FenD	FEND-F	5′-GGCCCGTTCTCTAAATCCAT-3′	269
		FEND-R	5′-GTCATGCTGACGAGAGCAAA-3′	
	FenA	FENA-F	5′-TGGATGGTTCCTCCGCATCTA-3′	
		FENA-R	5′-GGTGACGACCGCGCATTTTATT-3′	
Bacillomycin	BamC	Bacc1-f	5′-GAAGGACACGGAGAGAGTC-3′	875
		bacc1-r	5′-CGCTGATGACTGTTCATGCT-3′	
	bmyB	BMYB-F	5′-GAATCCCGTTGTTCTCCAAA-3′	370
		BMYB-R	5′-GCGGGTATTGAATGCTTGTT-3′	

### 2.4 Culture condition for lipopeptides production and extraction

The bacterial strains were cultured in 2 L flasks containing 500 mL of an optimized medium (OM) designed to enhance bioactive compound production. The OM composition (g/L) included peptone (20), sucrose (25), yeast extract (4.5), KH₂PO₄ (2), MnSO₄ (0.006), and MgSO₄ (0.6), consistent with formulations used to promote lipopeptides production in *Bacillus* species (Datta et al., 2018; Liu et al., 2021; Gharsallah et al., 2025). Flasks were incubated at 30°C for 5 days on an orbital shaker at 150 rpm, conditions known to maximize yield (Youssef et al., 2004; Habib et al., 2020; Gharsallah et al., 2025).

To extract extracellular proteins, the culture medium was centrifuged at 6000 rpm for 20 min to separate cells from the supernatant. Lipopeptides were precipitated by adjusting the supernatant pH to 2.0 using 6 N HCl, a method validated for effective precipitation (Vigneshwaran et al., 2021; Gharsallah et al., 2025). After overnight storage at 4°C, samples were centrifuged (6,000 rpm, 4°C) for 10 min. The precipitate was resuspended in distilled water, neutralized to pH 7.0 with 1 N NaOH to preserve bioactivity, (Gharsallah et al., 2025) and lyophilized for storage at −20°C.

### 2.5 Determination of anti-fungal activity

#### 2.5.1 Dual culture assay

The antifungal activity was assessed using the dual culture method on Potato Dextrose Agar medium. Mycelial disks (5 mm in diameter) from 6-day-old fungal cultures were positioned on one side of the plate, and a thin line of each bacterial isolate was streaked on the opposite side. A negative control, consisting solely of phytopathogenic fungi, was also prepared. The plates were incubated at 25°C until the control plates were fully colonized. The inhibition of pathogen growth was calculated using the formula: PI (%) = [(*D* − *d*)/*D*] × 100, where *D* represents the diameter of pathogen growth in control plates (mm), and *d* is the diameter of pathogen growth in test plates (mm) (Erdogan and Benlioglu, 2010).

#### 2.5.2 Fungal inhibition by bacterial extracted crude lipopeptides

The antifungal activity of the lipopeptides was evaluated using a radial diffusion assay. A 5 mm in diameter mycelial disc was placed at the center of Potato Dextrose Agar-containing Petri dish. Then, 20 μL of lipopeptide extract solution (100 mg/L) was carefully pipetted onto each disc. The Petri dishes were kept at 4°C for 4 h and then incubated at 25°C for 5 days. All experiments were performed in triplicate. The percentage of fungal growth inhibition was calculated using the same formula as in the dual culture assay.

#### 2.5.3 Inhibition of the fungal pathogen growth by volatile organic compounds

The antifungal activity of volatile organic compounds (VOCs) was assessed using the sandwich plate method. A 5 mm plug from a fresh fungal culture was placed in the center of a Potato Dextrose Agar-containing Petri dish. A second Petri dish, containing Luria-Bertani agar, was inoculated with a bacterial suspension and inverted over the first dish. The two plates were sealed with parafilm. Control plates without bacteria were also prepared. The experiment was conducted in triplicate, and all plates were incubated at 25°C until the control plates were fully colonized. The percentage of pathogen growth inhibition was calculated using the same formula as before. To confirm the role of VOCs, activated charcoal was added to absorb them, which eliminated the antifungal activity, leading to fungal growth similar to the control.

### 2.6 Data analysis

The obtained data were analyzed using SAS (Statistical Analysis System, version 9.4, SAS Institute Inc., Cary, NC, United States). The study employed three experimental replicates for all analyses. An analysis of variance (ANOVA) was performed using the GLM (general linear model) procedure to assess the effects of the factors PATHFUNG (fungal strains) and ANTAGSTRA (antagonistic strains), as well as their interaction, on the dependent variables INHIB (dual culture), LIPOPEP (LP inhibition), and VOCS (volatile organic compounds).

Differences between means were tested using the least significant difference (LSD) test with a significance threshold of *p* < 0.05. *R*-squared values were calculated to evaluate the proportion of variance explained by the models. Results were presented in tables and graphs, with *F* and *p*-values indicating the significance of main effects and interactions.

## 3 Results and discussion

### 3.1 Screening for genes involved in antibiotic biosynthesis

Lipopeptides (LPs) disrupt fungal membranes via pore formation (Ongena and Jacques, 2008). LPs produced by *Bacillus* species are categorized into three main families: surfactins, iturins, and fengycins. Their biosynthesis is regulated by non-ribosomal peptide synthetases (NRPSs), which influence both the quantity and structural diversity of LPs (Płaza et al., 2015). In this study, PCR-based gene detection was performed to identify key biosynthetic genes for surfactins, iturins, and fengycins in selected *Bacillus* strains (Table 2).

**Table 2:** PCR detection of biosynthetic genes involved in lipopeptide production in *Bacillus* species using specific primers.

	Metabolites									
	Surfactin		Iturin			Fengycin			Bacillomycin	
Strains	SurfA	Surf P	Itu C	Itu D	Itu D′	Feng A	Feng B	Feng D	Bam C	Bam B
*Bacillus velezensis* (H6)	+	−	+	+	+	+	−	+	−	+
*Bacillus subtilis* (S15)	+	−	−	+	+	+	−	+	−	+
*Bacillus cereus* (S32)	+	−	+	+	−	+	−	+	−	+
*Bacillus subtilis* (S40)	+	−	−	−	+	+	−	+	−	+

The detection of *fenA* and *fenD* genes in strains H6, S40, S32, and S15 indicates the potential for fengycin production. *fenD* is responsible for incorporating the third and fourth amino acids, while *fenA* encodes an NRPS module that integrates proline, glutamine/glutamic acid, and tyrosine (Guo and Yu, 2014). Sequence analysis confirmed >99% homology among strains and 97% identity with *Bacillus subtilis* plipastatin synthetase (GenBank: SRCM102747). However, the absence of *fenB*, which is essential for fengycin cyclization, could affect the final assembly and antifungal efficacy. This genetic variability is consistent with previous reports on strain-specific differences in fengycin production (Yasmin et al., 2022; Zeng et al., 2021).

The *ituD* gene, essential for iturin biosynthesis, was detected in all strains, showing 99% homology and 100% sequence identity with *B. amyloliquefaciens* WPS4-1 (GenBank: KY087954.1). Additionally, the *ituC* gene, involved in peptide elongation, was identified in S2 and S40, with sequences showing >95% similarity to *B. subtilis* and *B. amyloliquefaciens*. The presence of these genes suggests that these strains have the potential to produce iturins, which are known for their strong antifungal properties.

PCR screening did not detect the *sfp* gene, a key component of surfactin biosynthesis, in any of the tested isolates. This suggests either the absence or inactivation of the *sfp* gene, which could limit surfactin production. Further analysis of other genes within the surfactin operon (*srfAB*, *srfAC*, *srfAD*) is required to determine whether these strains can synthesize surfactins.

### 3.2 Screening of antifungal activity against phytopathogenic fungi

**Figure 1:**
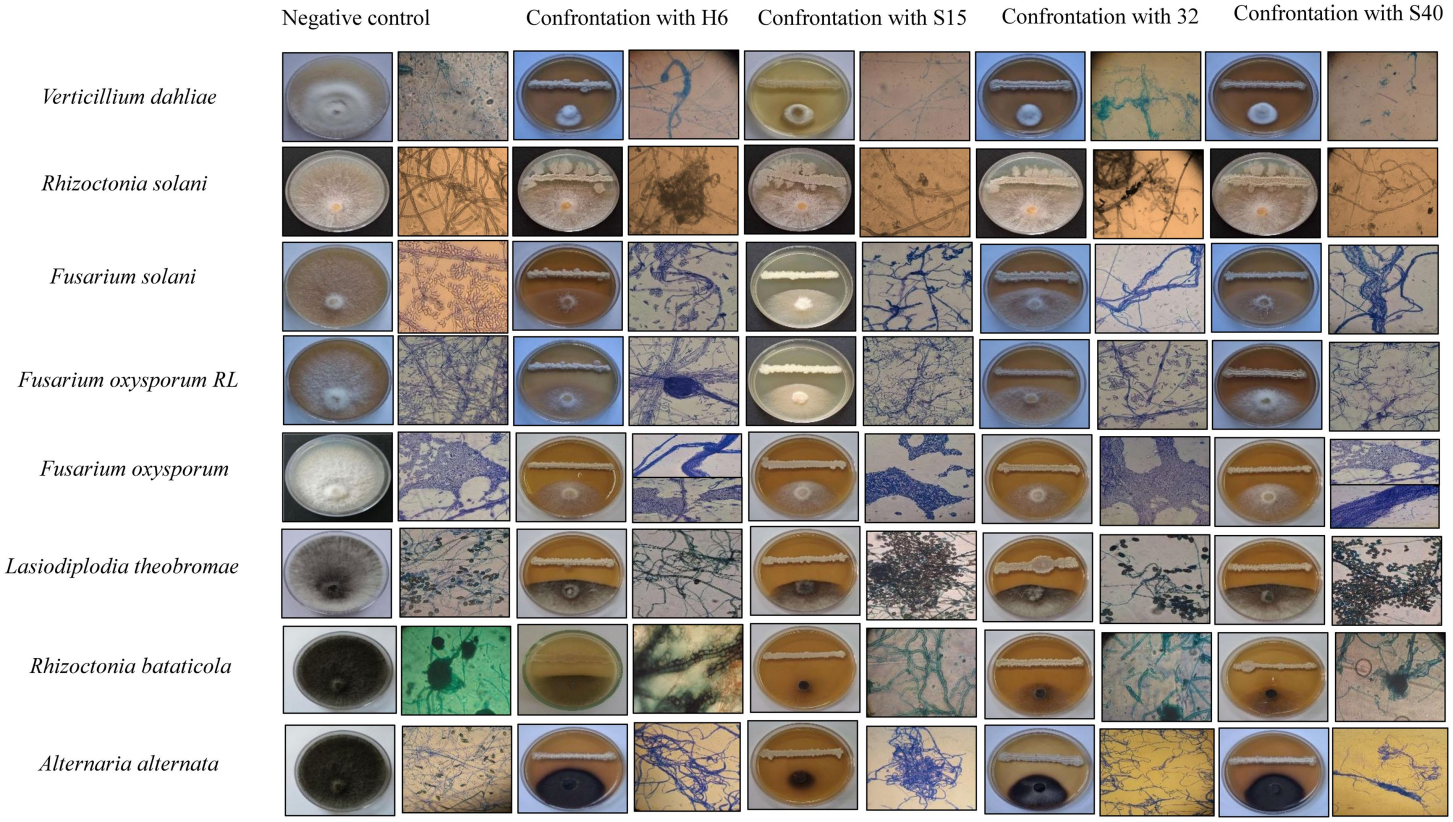
Growth inhibition of fungal phytopathogens by *Bacillus* strains using the dual-culture confrontation method, after 5 days of incubation, as well as observed under an optical microscope.

**Figure 2:**
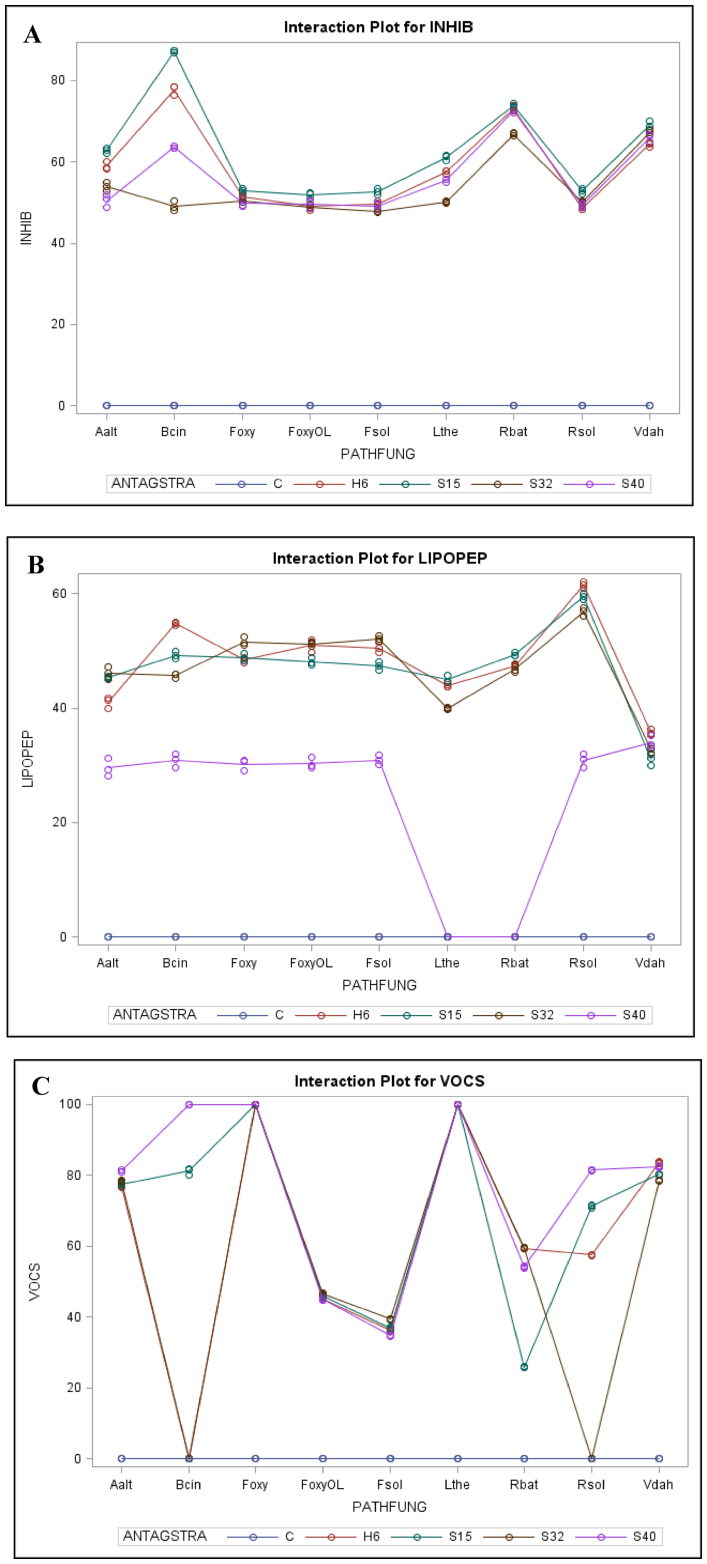
Interaction plot showing inhibitory effects. **(A)** Interaction plot illustrating the inhibitory effect of antagonistic strains (ANTAGSTRA) against pathogenic fungal species (PATHFUNG), using the dual culture assay. **(B)** Interaction plot depicting the inhibitory effect of extracted lipopeptides (LIPOPEP) from antagonistic strains on pathogenic fungal species (PATHFUNG). **(C)** Interaction plot showing the inhibitory effect of volatile organic compounds (VOCs) on pathogenic fungal species (PATHFUNG).

**Figure 3:**
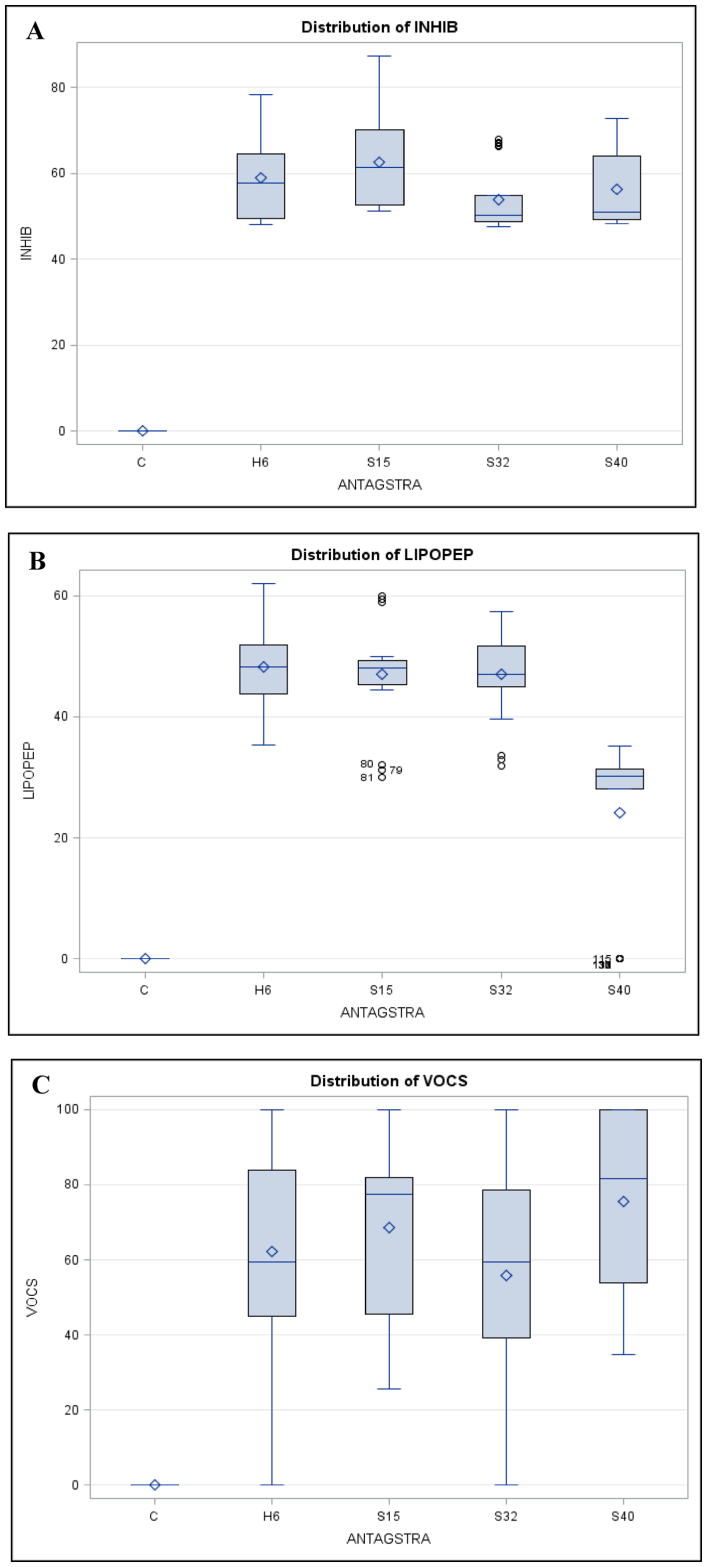
Comparative box plot of inhibitory activities. **(A)** The distribution of inhibitory effects of antagonistic strains (ANTAGSTRA) on pathogenic fungi. **(B)** Inhibitory activities of extracted lipopeptides (LIPOPEP) against pathogenic fungi. **(C)** Variation in inhibitory effect of VOCs among different antagonistic strains. H6, S15, and S32 as significantly different from the control.

The *Bacillus* strains in the current study (H6: *Bacillus velezensis*, S15: *Bacillus subtilis*, S32: *Bacillus cereus*, and S40: *Bacillus subtilis*) were specifically selected for their high lipopeptide production (Gharsallah et al., 2025). Lipopeptides are secondary metabolites known for their antifungal, antibacterial, and surfactant properties (Ongena and Jacques, 2008). These *Bacillus* spp. bacteria were screened for their antagonistic activity against *Fusarium oxysporum* (*F. oxy*), *Alternaria alternata* (*A. alt*), *Fusarium solani* (*F. sol*), *Fusarium oxysporum* f. sp. *radicis-lycopersici* (*FORL*), *Botrytis cinerea* (*B. cin*), *Verticillium dahliae* (*V. dah*), *Rhizoctonia solani* (*R. sol*), *Lasiodiplodia theobromae* (*L. the*), and *Rhizoctonia bataticola* (*R. bat*) using the dual-culture method (Figure 1). The results of the generalized linear model (GLM) analysis revealed considerable effects of antagonistic strains, crude lipopeptides, and VOCS (volatile organic compounds) on the dependent variable fungal inhibition. The bioactive compound generated by the H6, S15, S32, and S40 antagonistic strains demonstrated significant potential in inhibiting spoilage microorganisms, suggesting their application could extend the shelf life of perishable foods. Among the antifungal assays, 42% (45 out of 108) exhibited mycelial growth inhibition surpassing 60%. Notably, *Bacillus velezensis* H6 and *Bacillus subtilis* S15 demonstrated strong antifungal activity, reducing the growth of five fungal species (*A. alt*, *L. the*, *V. dah*, *R. bat*, and *B. cin*) by up to 60%. Particularly, the percentage inhibition (PI) values for *B. cin*, which reached 77.75% with H6 and 87.19% with S15 were the most noteworthy. These findings align with other studies that have demonstrated the efficacy of *Bacillus* lipopeptides against pathogenic fungi (Cawoy et al., 2011; Mnif et al., 2015; Cheffi et al., 2019). The General Linear Model (GLM) analysis for the dependent variable INHIB (fungal inhibition) revealed a highly significant model (*p* < 0.0001), with an *R*-squared value of 0.9995, indicating that 99.95% of the variance in inhibition was explained by the factors and their interactions (Figure 2). Mean comparisons, post-hoc tests, and least significant difference (LSD) tests revealed significant differences among treatments (Figure 3). The antagonistic strain S15 showed the highest mean inhibition value (PI = 62.67%), followed by H6 (PI = 58.91%). The high inhibition values were particularly associated with the pathogens *R. bataticola* (*R. bat*) and *B. cinerea* (*B. cin*), suggesting that these phytopathogens are highly susceptible to antagonistic treatments.

These results indicate that strains S15 and H6 likely produce metabolites or enzymes with strong antifungal activity, effectively inhibiting pathogen growth. This could include production of proteases, which degrade fungal cell wall proteins, leading to cell lysis, or secondary metabolites such as phenazines and cyclic lipopeptides, which may disrupt fungal respiration or spore germination (Paulitz and Bélanger, 2001; Raaijmakers et al., 2010; Loper et al., 2007). The findings highlight the potential of these antagonistic strains to produce bioactive compounds that target cellular integrity or metabolic pathways of pathogens, offering potential applications for biocontrol strategies in agriculture and food preservation.

### 3.3 Antifungal inhibition by lipopeptides extracted from *Bacillus* strains

**Figure 4:**
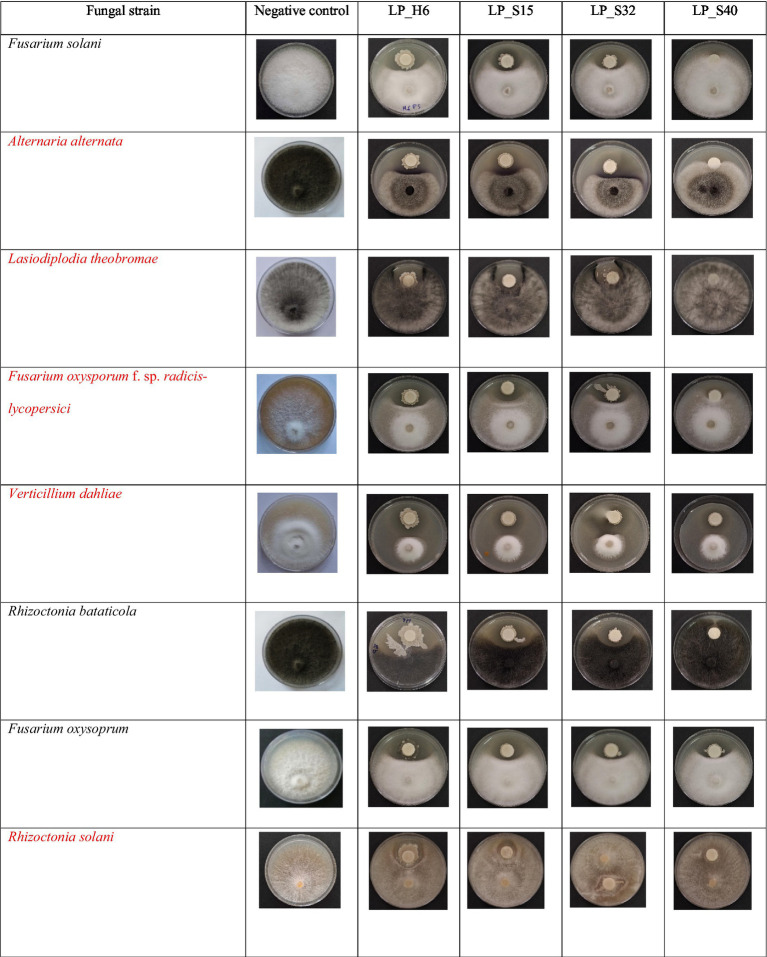
Inhibitory effect of *Bacillus*-derived lipopeptides on fungal growth.

The lipopeptide bioassay revealed slightly lower antifungal inhibition compared to the dual-culture assay (Figure 4). This difference may be due to the lack of direct bacterial-fungal interactions, such as colonization, nutrient competition, and the production of secondary metabolites (Ongena and Jacques, 2008). The lipopeptides from *Bacillus velezensis* (H6), *Bacillus subtilis* (S15), and *Bacillus cereus* (S32) showed strong antifungal activity, particularly against *Rhizoctonia solani* (*R. sol*), with 60% inhibition, which was higher than the 50% observed in the dual-culture assay. This suggests that lipopeptides contribute significantly to antifungal activity, likely through membrane-disrupting actions of compounds such as iturin, fengycin, and surfactin (Cawoy et al., 2011; Mnif et al., 2015). These mechanisms may explain the observed inhibition of fungal growth in the present study. According to Cawoy et al. (2014), bacterial strains that produce all three families of lipopeptides, or at least the iturin family, are more effective in inhibiting fungal growth. The simultaneous production of these antimicrobial metabolites enhances their ability to combat a wide range of pathogens (Han et al., 2018; Meena and Kanwar, 2015). The higher inhibition percentage observed for *R. sol* in the lipopeptide bioassay suggests that the extracted lipopeptides were either more concentrated or more effective in their purified form than during their *in situ* production in dual-culture assays. On the other hand, crude lipopeptides from *Bacillus subtilis* S40 showed the weakest antifungal activity, with no inhibition against *Lasiodiplodia theobromae* (*L. the*) and *Rhizoctonia bataticola* (*R. bat*). This underscores the strain-specific differences in lipopeptide production within *Bacillus subtilis*, as previous studies by Ongena and Jacques (2008) and Shafi et al. (2017) have shown that the antifungal efficacy of *Bacillus* strains depends on both the type and quantity of lipopeptides they produce. The lack of activity in S40 may be attributed to the absence or low concentration of specific lipopeptides effective against these particular fungi. The highest lipopeptide production was observed against *R. sol*, with an average inhibition of 41.73% across the phytopathogenic groups. The antagonistic strains had a highly significant effect on fungal growth (*F*-value = 26333.5, *p* < 0.0001) (Figures 2, 3). *Bacillus velezensis* H6 showed the highest mean inhibition (48.20%), closely followed by *Bacillus subtilis* S15 (47.07%). These antagonistic strains could potentially be combined to enhance lipopeptide production and improve antifungal activity against target pathogens. Lipopeptides may disrupt the pathogen’s membrane by increasing its permeability, leading to ion leakage and cell death. The high activity of strains H6 and S15 suggests the possible upregulation of surfactin, fengycin, or iturin-like compounds, which inhibit pathogen adhesion to host surfaces and biofilm formation, both of which are critical for pathogenicity (Stein, 2005; Ongena and Jacques, 2008).

### 3.4 Detection of antifungal activity of volatile organic compounds

**Figure 5:**
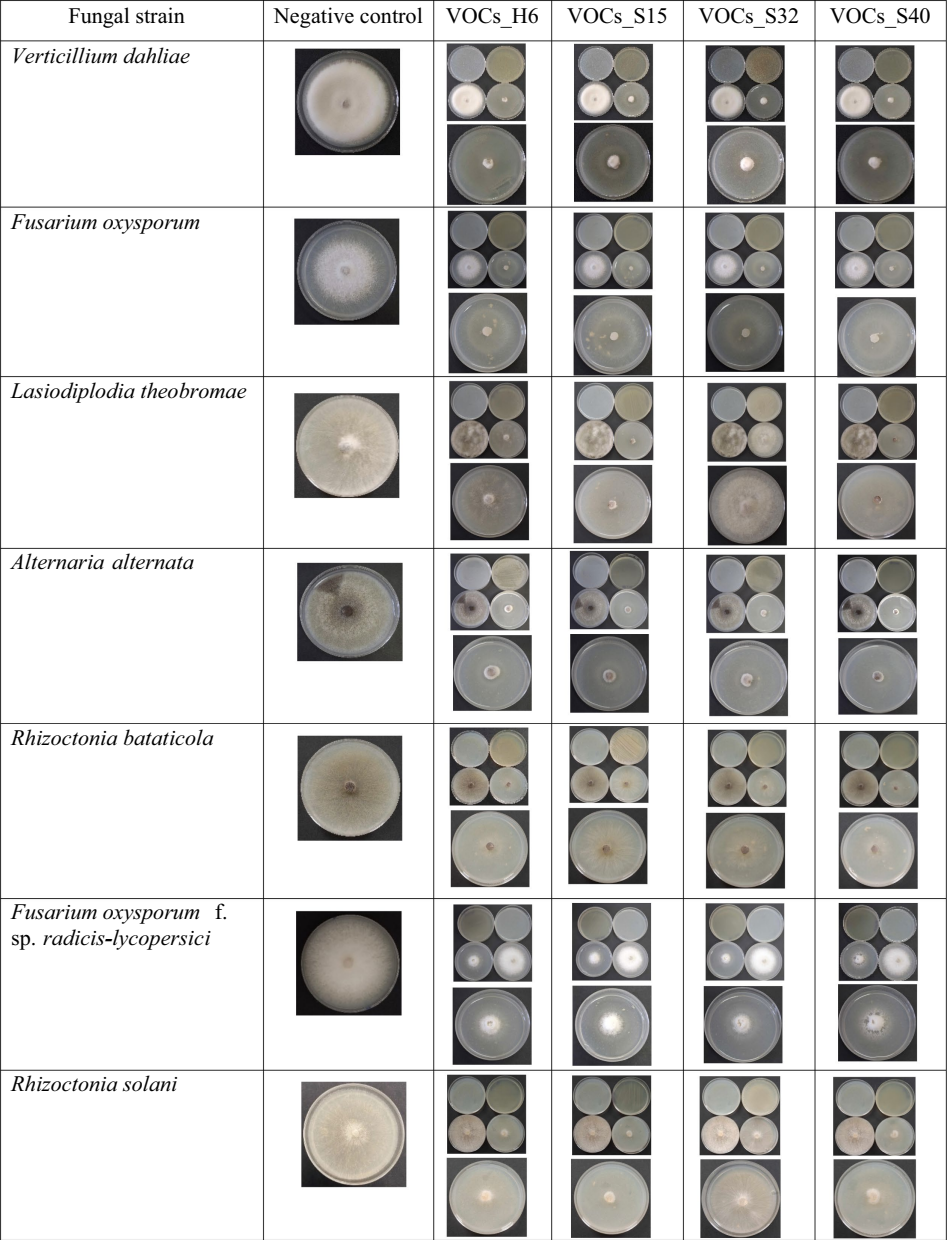
Antifungal activity of *Bacillus* VOCs against food spoilage fungi.

The sandwich plate technique (Figure 5) revealed that all tested *Bacillus* strains produced volatile organic compounds (VOCs) that inhibited the growth of *Fusarium oxysporum* and *Lasiodiplodia theobromae*, two major food spoilage fungi. These results are in line with previous studies that have highlighted the antifungal potential of VOCs produced by *Bacillus* species (Grahovac et al., 2023).

Interestingly, strain S40 exhibited the highest VOC-mediated inhibition, surpassing its antifungal effects observed in both dual-culture and lipopeptide assays. This dominance of VOC production in S40’s antagonistic activity aligns with findings by Caulier et al. (2019) and Audrain et al. (2015), who reported similar patterns in *Bacillus subtilis* and *Bacillus amyloliquefaciens*, where VOCs serve as the main antifungal agents rather than direct contact or lipopeptide secretion. Differences in VOC efficacy among strains likely arise from variations in metabolic pathways, substrate utilization, and environmental conditions, factors highlighted by Schulz-Bohm et al. (2017) as key influences on VOC production. Furthermore, the superior inhibition by VOCs compared to crude lipopeptides suggests that these gaseous metabolites may provide broader antifungal activity by effectively penetrating fungal structures, unlike direct-contact antimicrobial compounds (Chaves-López et al., 2015). This characteristic highlights the potential of VOC-producing *Bacillus* strains for biocontrol applications, especially in post-harvest disease management, where direct microbial interactions are limited. The VOCs produced by these strains offer a promising approach for food biopreservation, as they inhibit the growth of molds and pathogenic fungi responsible for food spoilage. The sandwich plate technique demonstrated that all *Bacillus* strains produced VOCs that completely inhibited (100% inhibition) the growth of *F. oxysporum* and *L. theobromae*.

The antifungal efficacy of VOCs, especially those produced by *Bacillus subtilis* and *Bacillus velezensis*, has been extensively documented in food preservation studies as demonstrated by Zhao et al. (2019) and Wang D. et al. (2022).

Unlike chemical fungicides, which can be toxic and promote pathogen resistance, VOCs from *Bacillus* strains provide a natural, sustainable, and residue-free alternative. Their gaseous nature enables them to diffuse through packaging materials and protect food products without direct contact, making them particularly effective for post-harvest preservation of perishable fruits and vegetables such as strawberries, grapes, and tomatoes (Chaves-López et al., 2015). Additionally, combining VOCs with other preservation methods, such as antimicrobial films or polysaccharide-based coatings, can enhance their efficacy by slowing fungal growth and extending the shelf life of perishable foods (Grahovac et al., 2023).

The significant VOC-mediated inhibition observed in *Bacillus subtilis* (S40), which surpassed its antifungal activity in dual-culture and lipopeptide assays, indicates that certain strains could be selectively utilized for food biopreservation. Incorporating VOC-producing *Bacillus* strains into active packaging or as a spray treatment for storage surfaces offers a promising strategy for natural food protection against mold and fungal pathogens. The high VOC production observed in S40 indicates potent antifungal activity, highlighting its potential application in controlling phytopathogens in agricultural settings (Weisskopf et al., 2021). The model for VOC production displayed an exceptionally high *R*-square value of 0.99997, indicating an almost perfect fit (Figures 2, 3). The analysis shows that different levels of antagonistic strains have varying impacts on VOCS. The antagonistic strains H6, S15, S32, and S40 had a highly significant effect (*F*-value = 443,789, *p* < 0.0001), with S40 and S15 showing the highest mean VOC production (75.44 and 68.74%, respectively). These strains could be combined to enhance VOC production, thereby improving their antimicrobial activity. The interaction between phytopathogenic fungi and antagonistic strains was significant (*F*-value = 25513.5, *p* < 0.05), indicating that VOC production plays a critical role in fungal inhibition. Notably, *F. oxysporum* and *L. theobromae* were completely inhibited by H6, S15, S32, and S40 (PI: 100%), suggesting that these combinations could be used to develop natural preservatives for food products.

The significant interaction effects between pathogenic fungi and antagonistic strains highlight potential synergistic or antagonistic relationships (Figures 2, 3). Interaction plots reveal distinct patterns, showing that each pathogen responds differently to various antagonists. For example, combinations involving *R. bataticola* (*R. bat*) and antagonistic strains S15 or H6 led to significantly higher levels of fungal inhibition in both dual-culture and bacterial-extracted lipopeptide assays. This suggests that these pairings of antagonistic strains may induce co-production of inhibitory pathways, where the presence of certain antagonists enhances the production of antifungal compounds. This phenomenon could be explained by the cross-induction of biosynthetic gene clusters, known to regulate the production of secondary metabolites such as lipopeptides and VOCs. The strains S15 and H6 may activate specific genetic pathways in *R. bat* or other pathogens, leading to increased synthesis of bioactive compounds that inhibit fungal growth. These findings align with research by Han et al. (2018), which emphasizes the complex microbial interactions that enhance the production of antimicrobial metabolites. Additionally, studies by Raaijmakers et al. (2010) and Loper et al. (2007) support the role of cyclic lipopeptides and phenazines in disrupting fungal cellular integrity and metabolic pathways.

These insights underscore the importance of strain-specific interactions in developing effective biocontrol strategies. The combination of specific pathogens and antagonists can significantly influence the production of inhibitory compound, helping optimize the use of microbial antagonists for sustainable agriculture and food preservation.

## 4 Conclusion

In this study, strains H6 and S15 demonstrated the highest levels of fungal inhibition, highlighting their potentiel applications in food preservation and biocontrol. Lipopeptides, especially iturins and fengycins, were instrumental in reducing fungal growth, while volatile organic compounds (VOCs) exhibited complete inhibition of specific pathogens. Additionally, genetic analysis revealed the presence of essential biosynthetic genes responsible for antifungal compound production, reinforcing the suitability of these strains for agricultural and food preservation purposes.

## Data Availability

The datasets presented in this study can be found in online repositories. The names of the repository/repositories and accession number(s) can be found in the article/Supplementary material.

## Footnotes

^1^ http://www.ncbi.nlm.nih.gov/

## Supplementary material

SUPPLEMENTARY FIGURE 1 PCR detection of biosynthetic genes involved in lipopeptide production in *Bacillus* species using specific primers. **(A)** Strain H6 (*Bacillus velezensis*). **(B)** Strain S15 (*Bacillus subtilis*). **(C)** Strain S32 (*Bacillus cereus*), and **(D)** Strain S40 (*Bacillus subtilis*).

SUPPLEMENTARY FIGURE 2 Sequences of PCR-amplified genes involved in lipopeptide biosynthesis in *Bacillus* species using specific primers.
