# Supplementary figures and images for: Antifungal potential of Bacillus strains: implications for biocontrol strategies in food safety and sustainable agriculture
Source: Front Microbiol. 2025 Jul 15;16:1615252. doi: 10.3389/fmicb.2025.1615252 (PMC12303991; doi:10.3389/fmicb.2025.1615252)

Supplementary Figure 1:

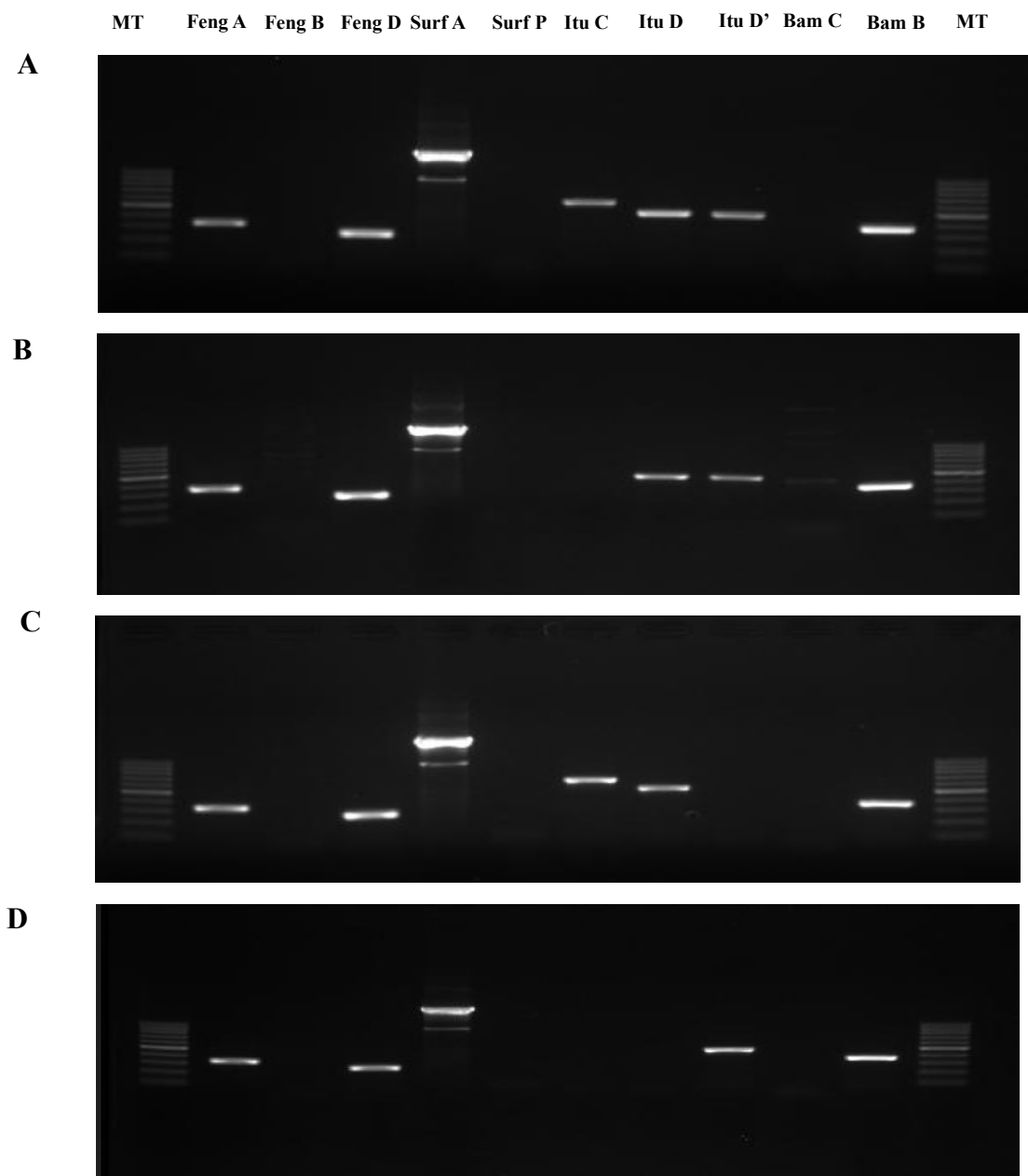

Supplement: SUPPLEMENTARY FIGURE 1 — PCR detection of biosynthetic genes involved in lipopeptide production in Bacillus species using specific primers. (A) Strain H6 (Bacillus velezensis). (B) Strain S15 (Bacillus subtilis). (C) Strain S32 (Bacillus cereus), and (D) Strain S40 (Bacillus subtilis). [file Data_Sheet_1.pdf]
