# Supplementary material for: Antifungal potential of Bacillus strains: implications for biocontrol strategies in food safety and sustainable agriculture
Source: Front Microbiol. 2025 Jul 15;16:1615252. doi: 10.3389/fmicb.2025.1615252 (PMC12303991; doi:10.3389/fmicb.2025.1615252)
Supplement: SUPPLEMENTARY FIGURE 2 — Sequences of PCR-amplified genes involved in lipopeptide biosynthesis in Bacillus species using specific primers. [file Data_Sheet_2.pdf]

Supplementary Figure 2:

```
                   10        20        30        40        50        60        70        80
H6   FenA   AAWAMGGACGGGCACCCGGAACGGGTTTACCAATCGGAAGCCGCGTCCGTCCGCTGTCCCGTTTTCGGTCACAGACGTAA 80
S15  FenA   -ACGACGAT-GGCACCCGGAACGGGTTTACCAATCGGAAGCCGCGTCCGTCCGCTGTCCCGTTTTCGGTCACAGACGTAA 78
S32  FenA   AACTAMGAC-GGGCACCCGGACGGGTTTACCAATCGGAAGCCGCGTCCGTCCGCTGTCCCGTTTTCGGTCACAGACGTAA 79
S40  FenA   ----CTGACTGGCACCCGGAACGGGTTTACCAATCGGAAGCCGCGTCCGTCCGCTGTCCCGTTTTCGGTCACAGACGTAA 76

                   90       100       110       120       130       140       150       160
H6   FenA   AACGCCGCATCMACCGTCGCTTCCGTCGGCCCRTAGCCGTGAATAAGCAYSGTCTCCGGCARMAGAKCKGCAAACCGGGC 160
S15  FenA   AACGCCGCATCMACCGTCGCTTCCGTCGGCCCRTAGCCGTGAATAAGCAYSGTCTCCGGCARMAGAKCKGCAAACCGGGC 158
S32  FenA   AACGCCGCATCMACCGTCGCTTCCGTCGGCCCRTAGCCGTGAATAAGCAYSGTCTCCGGCARMAGAKCKGCAAACCGGGC 159
S40  FenA   AACGCCGCATCMACCGTCGCTTCCGTCGGCCCRTAGCCGTGAATAAGCAYSGTCTCCGGCARMAGAKCKGCAAACCGGGC 156

                  170       180       190       200       210       220       230       240
H6   FenA   AGCCGTAAGCGGKGAAAGAGCTTCSCCTCCGGCAAACACGCGTGTCARKGTTCTGCCGAGGCGTGTTCTTGTTTCCKCAG 240
S15  FenA   AGCCGTAAGCGGKGAAAGAGCTTCSCCTCCGGCAAACACGCGTGTCARKGTTCTGCCGAGGCGTGTTCTTGTTTCCKCAG 238
S32  FenA   AGCCGTAAGCGGKGAAAGAGCTTCSCCTCCGGCAAACACGCGTGTCARKGTTCTGCCGAGGCGTGTTCTTGTTTCCKCAG 239
S40  FenA   AGCCGTAAGCGGKGAAAGAGCTTCSCCTCCGGCAAACACGCGTGTCARKGTTCTGCCGAGGCGTGTTCTTGTTTCCKCAG 236

                  250       260       270       280       290       300
H6   FenA   GCTCYGTTTCCAGCGCGTCTAAAAAGCTGTTGACCATYGCCGGAATAAAATGCGCGKTYCGTCACCSA 308
S15  FenA   GCTCYGTTTCCAGCGCGTCTAAAAAGCTGTTGACCATYGCCGGAATAAAATGCGCGGTC-GTCACCSA 305
S32  FenA   GCTCYGTTTCCAGCGCGTCTAAAAAGCTGTTGACCATYGCCGGAATAAAATGCGCGGTYCGTCACWCA 307
S40  FenA   GCTCYGTTTCCAGCGCGTCTAAAAAGCTGTTGACCATYGCCGGAATAAAATGCGCGTYCSGTCACCSA 304


                   10        20        30        40        50        60        70        80
H6   FenD   ACCGTACATCATGATGCCTTGCGCCTTGTCTGCATAAAAGACGAAGA-GAAAGGTCTTCTCCTCTTCAACAGACCGGCTG 79
S15  FenD   ACCGTACATCATGATGCCTTGCGCCTTGTCTGCATAAAAGACGAAGA-GAAAGGTCTTCTCCTCTTCAACAGACCGGCTG 79
S32  FenD   ACCGTACATCATGATGCCTTGCGCCTTGTCTGCATAAAAGACGAAGA-GAAAGGTCTTCTCCTCTTCAACAGACCGGCTG 79
S40  FenD   ACCGTACATCATGATGCCTTGCGCCTTGTCTGCATAAAAGACGAAGWAGAAAGGTCTTCTCCTCTTCAACAGACCGGCTG 80

                   90       100       110       120       130       140       150       160
H6   FenD   ACCTCGCAGATGAACAGCTTTACAGCCTGACCATATTAGAAATGGAAGGCGATGAGCATGAGAAGGAACGTTTTGTAAAA 159
S15  FenD   ACCTCGCAGATGAACAGCTTTACAGCCTGACCATATTAGAAATGGAAGGCGATGAGCATGAGAAGGAACGTTTTGTAAAA 159
S32  FenD   ACCTCGCAGATGAACAGCTTTACAGCCTGACCATATTAGAAATGGAAGGCGATGAGCATGAGAAGGAACGTTTTGTAAAA 159
S40  FenD   ACCTCGCAGATGAACAGCTTTACAGCCTGACCATATTAGAAATGGAAGGCGATGAGCATGAGAAGGAACGTTTTGTAAAA 160

                  170       180       190       200       210
H6   FenD   CGCCGCGTCGCAGAGCTTCAGAGAAACATGGATTTAGAGAACGGGCCCA-- 208
S15  FenD   CGCCGCGTCGCAGAGCTTCAGAGAAACATGGATTTAGGAAACGGGCCSA-- 208
S32  FenD   CGCCGCGTCGCAGAGCTTCAGAGAAACATGGATTTAGAGAACGGGCCCA-- 208
S40  FenD   CGCCGCGTCGCAGAGCTTCAGAGAAACATGGATTTAGAAARGGGGCMRAAT 211


                   10        20        30        40        50        60        70        80
H6   ItuD   ----------ATTTCAAATGTCACCGGCGGCCCTTACAGCAGCGGAAATTCGATCAGCGAACATCTCAAGCAGCACATGA 70
S15  ItuD   ATGGCCGATCATTTCAAATGTCACCGGCGGCCCTTACAGCAGCGGAAATTCGATCAGCGAACATCTCAAGCAGCACATGA 80
S40  ItuD   ATGGCCGATCATTTCAAATGTCACCGGCGGCCCTTACAGCAGCGGAAATTCGATCAGCGAACATCTCAAGCAGCACATGA 80

                   90       100       110       120       130       140       150       160
H6   ItuD   CGATGCCGGTTAGATGGACAGAATCGATGCATTACTTGCTTTTACATAGAATCACAGAAGTCATCGAAATGGGTCCGAAC 150
S15  ItuD   CGATGCCGGTTAGATGGACAGAATCGATGCATTACTTGCTTTTACATAGAATCACAGAAGTCATCGAAATGGGTCCGAAC 160
S40  ItuD   CGATGCCGGTTAGATGGACAGAATCGATGCATTACTTGCTTTTACATAGAATCACAGAAGTCATCGAAATGGGTCCGAAC 160

                  170       180       190       200       210       220       230       240
H6   ItuD   AATGTCTTATCCGGTCTGCTGAGAAAAACAACGAATCACATTGTACCTTATCCCTTAGGACAGACATCTGATGTTCCCCC 230
S15  ItuD   AATGTCTTATCCGGTCTGCTGAGAAAAACAACGAATCACATTGTACCTTATCCCTTAGGACAGACATCTGATGTTCCCCC 240
S40  ItuD   AATGTCTTATCCGGTCTGCTGAGAAAAACAACGAATCACATTGTACCTTATCCCTTAGGACAGACATCTGATGTTCCCCC 240

                  250       260       270       280       290       300       310       320
H6   ItuD   GCTTTCCAATCCAGCGGAAAGAAAGAAACATATTGTCCATTTACGCAAAAAACAACTGAATAAATTGATGATTCAATCCG 310
S15  ItuD   GCTTTCCAATCCAGCGGAAAGAAAGAAACATATTGTCCATTTACGCAAAAAACAACTGAATAAATTGATGATTCAATCCG 320
S40  ItuD   GCTTTCCAATCCAGCGGAAAGAAAGAAACATATTGTCCATTTACGCAAAAAACAACTGAATAAATTGATGATTCAATCCG 320

                  330       340       350       360       370       380
H6   ItuD   TCATTGCGCGAAATTACAACAAGGATTCAGCGGCTTATTCCAATATGACGACGCCATTTTTT- 372
S15  ItuD   TCATTGCGCGAAATTACAACAAGGATTCAGCGGCTTATTCCAATATGACGACGCCATATTTTT 383
S40  ItuD   TCATTGCGCGAAATTACAACAAGGATTCAGCGGCTTATTCCAATATGACGACGCCATTTTTT- 382
```

```
                 10        20        30        40        50        60        70        80
H6   ItuC   --CTGAAAM-CATTCACTTCTCGTTGACAATCAAACTGCGAAAACTGAATATCCGCGGGATAAGACGGTTTATCAGTTAT 77
S40  ItuC   ATCGGAGACACATTCACTTCTCGTTGACAATCAAACTGCGAAAACTGAATATCCGCGGGATAAGACGGTTTATCAGTTAT 80

                 90       100       110       120       130       140       150       160
H6   ItuC   TCGAAGAACAGATGAAACGAACACCGGATCAAGCAGCCGTTATTTACGGAGAAAAGCAATTCACATATCGTCAGCTCAAT 157
S40  ItuC   TCGAAGAACAGATGAAACGAACACCGGATCAAGCAGCCGTTATTTACGGAGAAAAGCAATTCACATATCGTCAGCTCAAT 160

                170       180       190       200       210       220       230       240
H6   ItuC   GAACGTGCCAATCAATTAGCCCGAACGTTAAGGAAAAAGGGGGTAAAGACGGATCGGCTCACTGCGATTATCTGCGAACA 237
S40  ItuC   GAACGTGCCAATCAATTAGCCCGAACGTTAAGGAAAAAGGGGGTAAAGACGGATCGGCTCACTGCGATTATCTGCSAACA 240

                250       260       270       280       290       300       310       320
H6   ItuC   TGAGATCGAACTGGTCGTGGGAATACTGGCC-GTTTTAAAAGCGGGAGGGGCCTATGTGCCAATTGATCCGGATTATCCG 316
S40  ItuC   TGAGATCSAACTGGTCGTGGGRAWACTGGSCCGTTTTAAAAGCGGGAGGGGCCTATGTGCCAATTGATCCGGRWTWWYYS 320

                330       340       350       360       370       380       390       400
H6   ItuC   AAGCATCGCATACAGTATATAGTAGAAGACTCCCAAGCTGATATCGTCCTGACGCAGAGCCATCTTCAAAAACAGTTGGA 396
S40  ItuC   AAGCAWCGCATACAGTATATWGTARAARAMTCCCAAGCTGATATCGTCCTGACGCAGAGCCATCTTCAAAAACAGTTGGA 400

                410       420       430       440       450       460       470       480
H6   ItuC   ACTTGCGGGCACAATGGTTTTCCTCGATCAGGAAAGCTCTTACCACGAAGACGGCTCTTACCTGGAACCGATCAGCAGTA 476
S40  ItuC   ACTTGCGGGCACAATGGTTTTCCTCGATCAGGAAAGCTCTTACCACGAAGACGGCTCTTACCTGGAACCGATCAGCAGTA 480

                490       500       510       520       530       540       550
H6   ItuC   CGAAG-GATTTGGCCTATGTCATTTATACGTCAGGCTCCACAGGCAAGCCTAAAGGAGTGGCAATTGAGCATCAGTTT- 553
S40  ItuC   CGAAAAGGTWTGGCCTATGTCATTTATACGTCAGGCTCCACAGGCAAGCCTAAAGGAGTGGCAATTGAGCATCACTTTT 559
```
